# Union Rate on Hinge Side after Open-Door Laminoplasty Using Maxillofacial Titanium Miniplate

**DOI:** 10.1155/2013/767343

**Published:** 2013-11-26

**Authors:** Koopong Siribumrungwong, Theerasan Kiriratnikom, Boonsin Tangtrakulwanich

**Affiliations:** Department of Orthopedic Surgery and Physical Medicine, Faculty of Medicine, Prince of Songkla University, Hat Yai, Songkla 90110, Thailand

## Abstract

*Background*. One of the important complications of open-door laminoplasty is a premature laminoplasty closure. In order to prevent premature laminoplasty closure many techniques have been described and a titanium miniplate is one of the instruments to maintain cervical canal expansion. This study was performed to evaluate the effectiveness of titanium miniplates on the union rate for open-door laminoplasty. *Materials and Methods*. We performed open-door laminoplasty in 68 levels of fourteen patients using maxillofacial titanium miniplates. Axial computed tomography scans were obtained at 6 months postoperatively to evaluate the union rates of the hinge side. The Japanese Orthopedic Association (JOA) score was used to compare the clinical outcomes before and after surgery. *Results*. Computed tomography scan data was available on 68 levels in 14 patients. There were no premature closures of the hinge or miniplate dislodgements. The union rate on the hinge side was 70.5% (48/68). The mean JOA score increased significantly from 7.0 before surgery to 10.2, 12.2, and 13.0 after surgery at 1, 3, and 6 months, respectively. *Conclusion*. Open-door laminoplasty using maxillofacial titanium miniplates can provide union rates comparable to other techniques. It can maintain canal expansion without failures, dislodgements, and premature closures.

## 1. Introduction

Open-door laminoplasty is a standard procedure for the treatment of multiple levels of cervical spondylotic myelopathy and ossification of posterior longitudinal ligaments (OPLL). There are several techniques to maintain cervical canal expansion such as the Hirabayashi technique [1] which is the classic open-door laminoplasty that maintains cervical canal expansion by suturing to the contralateral soft tissue. The Itoh and Tsuji technique [2] maintains an elevated lamina by a spinous process spacer with suturing to the lateral mass. However, sutures do not provide a rigid fixation [3] and so premature laminoplasty closure is one of the important complications in open-door laminoplasty which results in subsequent restenosis. In one series using suturing techniques, patients developed some degree of reclosure at one or more levels up to 34% [4]. In order to prevent premature laminoplasty closure with a rigid fixation many techniques such as ceramic spacers, bone strut grafts, and plating systems have been introduced. However, bone struts and ceramic spacers are associated with the potential of graft dislodgement which can lead to reclosure of laminoplasty or neurological deficit if the spacers dislodge into the canal [5, 6]. Plating can provide immediate rigid fixation [7] and there are many types of plates used to maintain cervical canal expansion. In 1996 O'Brien et al. [8] used a maxillofacial miniplate and screws to maintain the canal expansion in an open-door laminoplasty. But this procedure is technically difficult as the assistant and primary surgeon must work together to hold the lamina and plate in place while drilling, tapping, and inserting the requisite screws [7]. A new plating design was developed [7] in an attempt to provide easier fixation. The results showed good effectiveness of the new plate design [9]. There were no plate failures or premature laminoplasty closures. The union rates after using the new plate design were 55%, 77%, and 93% at 3, 6, and 12 months, respectively. But the cost of the new laminoplasty plate is still high. The first advantage of the maxillofacial titanium miniplate over the commercial laminoplasty plate is the lower cost. The second advantage is when the patient has a bony anatomical variation or the open-door laminoplasty site does not match with the commercial laminoplasty plate the maxillofacial titanium miniplate can be adjusted to different lengths. This plate can also bend in all three dimensions to follow the contour of an abnormal anatomy on the open-door side. In our institution, the authors use a maxillofacial titanium miniplate to maintain cervical canal expansion in open-door laminoplasty. However, there are only a few studies evaluating its effectiveness on the union rate of the hinge side. The purpose of this study was to evaluate the effectiveness of the maxillofacial titanium miniplate to maintain cervical canal expansion in open-door laminoplasty.

## 2. Materials and Methods

This was a cohort study of 14 consecutive patients including 8 male and 6 female patients from April 2011 to February 2012 with 68 levels of open-door laminoplasty using maxillofacial titanium miniplates. This study was approved by the Institutional Review Board, Faculty of Medicine, Prince of Songkla University. All patients signed a written consent form before surgery and all patients had a radiographic proven multilevel myelopathy or OPLL on MRI and were indicated for open-door laminoplasty. Inclusion criteria were patients who were diagnosed as multilevel cervical spondylotic myelopathy or OPLL. Eligible patients were aged 40–80 years. All patients underwent open-door laminoplasty using a maxillofacial titanium miniplate to maintain cervical canal expansion at Songklanagarind Hospital. We excluded patients with a history of titanium allergy or spinal cord myelopathy from trauma.

**Figure 1:**
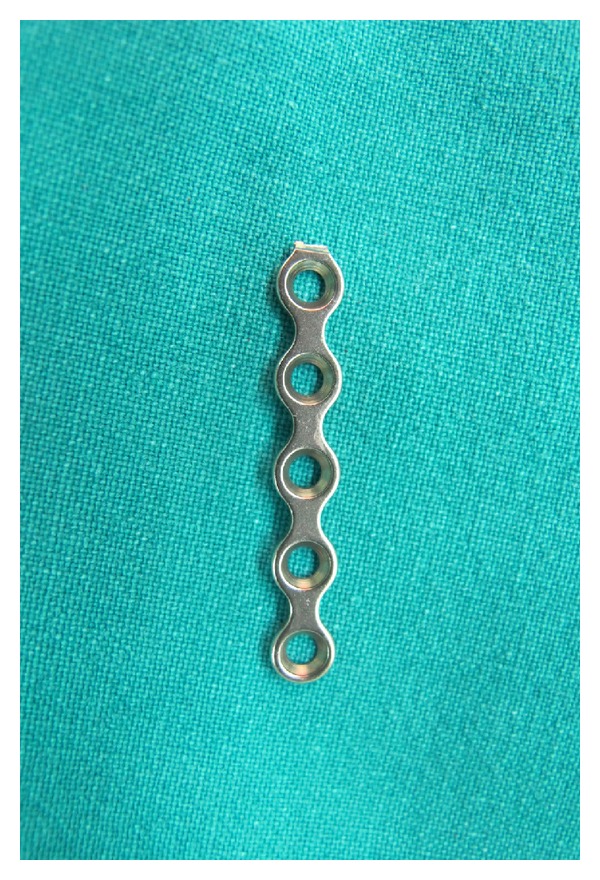
Maxillofacial titanium miniplate.

**Figure 2:**
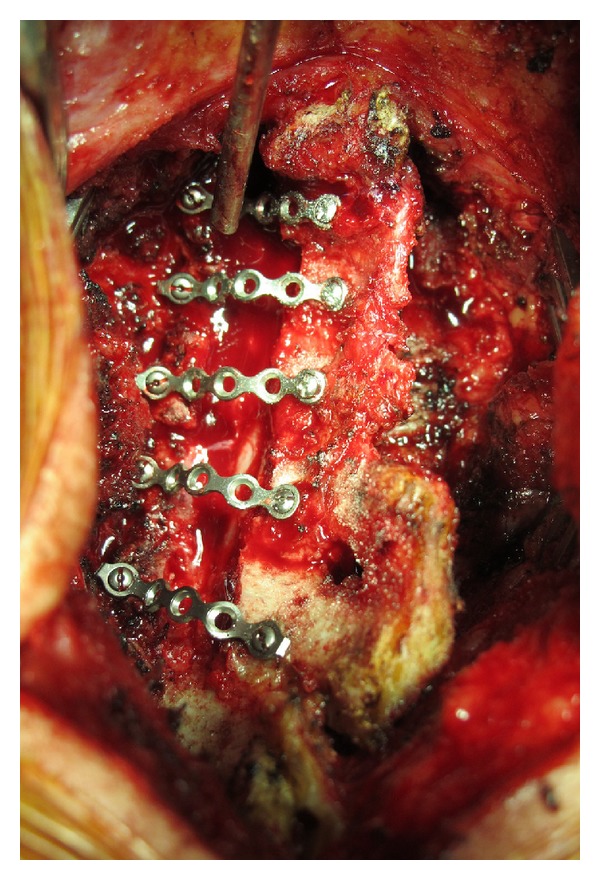
Five maxillofacial titanium miniplates were applied at decompressed levels.

**Figure 3:**
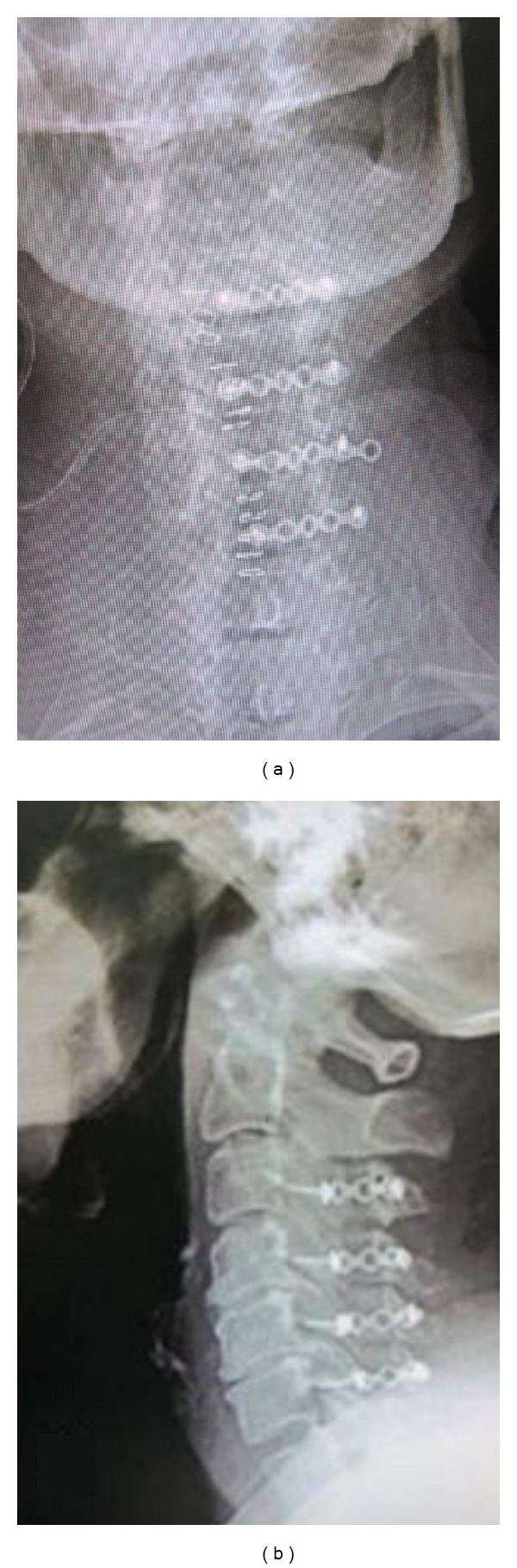
Postoperative AP and lateral film.

The patients underwent general anesthesia and the surgery was performed by an experienced spine surgeon. The patients were placed in a prone position with Mayfield traction to control the position of the cervical spine. We used a standard midline posterior approach. The number of levels that required decompression depended on the pattern of cord compression. In all cases, a 3 mm high speed burr was used to create the hinge at the junction between the lateral mass and laminar. The hinge side was not grafted. The open side was stabilized with the maxillofacial miniplate and miniscrews (Medtronic Sofamor Danek, Memphis, TN, USA) (Figure 1). The miniplates were cut into appropriate lengths and bent before application at the open-door side to maintain the decompressed spinal canal (Figure 2). We applied the miniplates at all decompressed levels (Figure 3). We did not add strut grafts at the open-door side. One screw was fixed on each side. One redivac drain was placed and removed 48 hours postoperatively.

### 2.1. Postoperative Rehabilitation

Immediately following the surgery, the patients were placed into a soft cervical collar. Although the soft collar did not provide adequate immobilization of the cervical spine, [12] we used the soft collar for resting the cervical spine muscle postoperatively. After discharge from the hospital, the patients were encouraged to engage in range-of-motion exercises of the cervical spine as permitted by wound pain. A soft collar was usually applied to all patients for 4–6 weeks and then removed once the wound pain subsided. Low-strength active resistive exercises were started after the wound pain subsided. A previous study by Assano et al. [13] showed a decrease in the incidence of axial neck symptoms using this active rehabilitation protocol.

**Figure 4:**
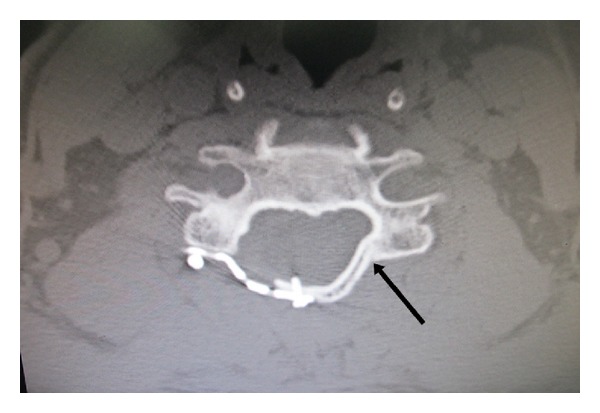
Union of hinge side as defined by the union of both dorsal and ventral cortices (arrow).

**Figure 5:**
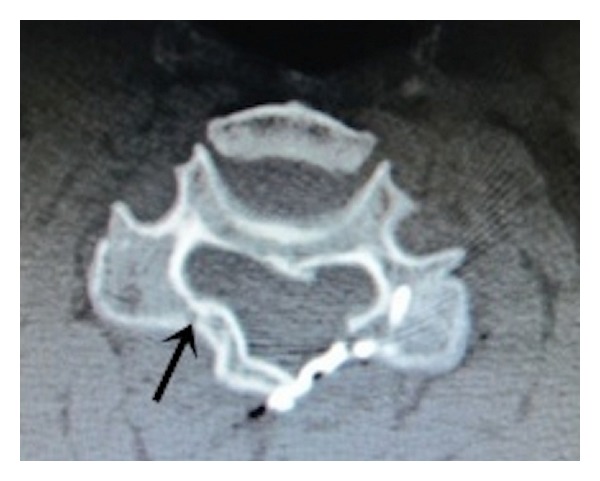
The hinge was perfectly formed continuously only at the ventral cortex that immediately appeared after surgery (arrow) that may cause a false positive on interpretation.

The clinical preoperative and postoperative outcomes were assessed by Japanese Orthopedic Association Scores [14] (JOA scores) at 1, 3, and 6 months. The recovery rate at 1, 3, and 6 months, operative time, and blood loss were also recorded. Reclosure of the lamina or the “spring-back” phenomenon usually occurs postoperatively before 6 months [15]. The union rate at 6 months postoperatively was documented by computed tomography scan (CT). The dorsal and ventral cortices of each hinge were assessed separately for the presence of either cancellous or cortical bone bridging. We defined the union of the hinge side by the presence of bridging bone of both the dorsal and ventral cortices (Figure 4). If only one cortical bridging was noted, the hinge was not defined as a union as we could see whether or not the hinge was perfectly formed as a continuous ventral cortex that immediately appears after surgery (Figure 5) [9]. We used specific criteria to avoid any false positive from an interpretation of the union on the hinge side. Complications such as plate dislodgement, postoperative C5 palsy, and reclosure of laminoplasty were also recorded.

### 2.2. Statistical Analysis

Analysis of variance (ANOVA) test was used to evaluate the statistically significant difference of union rates between levels. The *P* value was set at 0.05. The statistical analysis was calculated by SPSS program version 14.0.

## 3. Results

**Figure 6:**
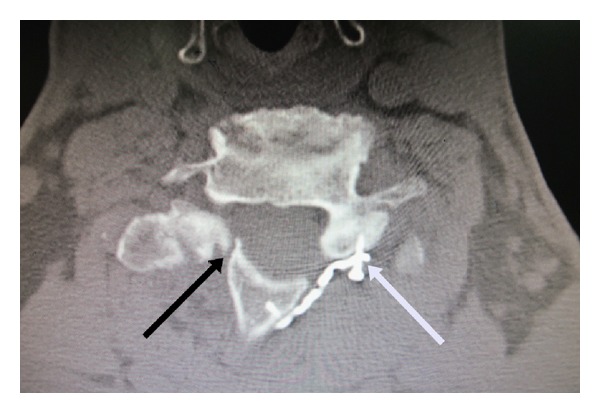
Axial CT scan at 6 months demonstrates screw back out (white arrow) and nonunion on the hinge side (black arrow) (no bone bridge at both dorsal and ventral cortices). There was no reclosure of laminoplasty.

There were 14 consecutive patients included in this study with 8 males and 6 females. Sixty-eight levels of open-door laminoplasty were involved using maxillofacial titanium miniplates. The mean age was 58 years ± 5.6. Twelve patients underwent surgery for five levels (C3–C7) and two patients for four levels (C3–C6). All patients received followups at 1, 3, and 6 months. CT scan data was available on 68 levels at 6 months. Mean operative time was 181 ± 36 minutes. Mean blood loss was 330 ± 82 cc. The mean JOA score increased significantly from 7.0 ± 1.0 before surgery to 10.21 ± 1.8, 12.21 ± 1.8, and 13.0 ± 1.3 after surgery at 1, 3, and 6 months, respectively. Mean recovery rates at 1, 3, and 6 months were 32.6%, 52.4%, and 60.15%, respectively. At 6 months the healing rate at C3, C4, C5, C6, and C7 were 71.4%, 64.2%, 64.2%, 71.4%, and 83.3%, respectively. There was no statistically significant difference in the union rates between each level. The mean percentage of all levels of hinges that formed a union at 6 months postoperatively from CT scans was 70.5%; however, there were no cases at any time which showed premature closure of the lamina, plate dislodgement, or broken plates even in the cases with a nonunion hinge (Figure 6). Two of 68 levels (2.9%) had evidence of loosening or reversing of screws on final postoperative follow-up radiographs, but no reclosure of the lamina occurred (Figure 6). Screw back-out was not associated with any neurological consequences. No one developed a cervical kyphotic deformity after surgery.

## 4. Discussion

Open-door laminoplasty is one of the most common decompressive procedures for addressing congenital canal stenosis, multiple levels of cervical spondylotic myelopathy, and OPLL. It can provide sufficient decompression of the cervical spine by providing sufficient space for the spinal cord to drift in a posterior direction and preserve motion of the cervical spine. There are a number of techniques used to maintain cervical canal expansion. However, currently there is no “best” technique that is recommended. Plating is one type of the fixation techniques that can provide immediate rigid fixation [7] and can allow early mobilization. In this study, we evaluated the results of the maxillofacial titanium miniplate in maintaining cervical canal expansion in open-door laminoplasty with specific criteria. We used the criteria of union based on both dorsal and ventral cortices as described by Rhee et al. [9]. These criteria can avoid a false positive from a perfectly hinged creation (bridging only the ventral cortex).

A new titanium plating design was developed [7] in an attempt to provide easier fixation [16]. But in our hospital the cost of the new titanium plate design is about 800 US dollars/level which is much more expensive than the maxillofacial titanium miniplate (70 US dollars/level). The cost of the new plating design is almost ten times more expensive than the maxillofacial titanium miniplate in levels. Our study of the maxillofacial titanium miniplate showed a union rate of 70.5% on the hinged side at 6 months. That is comparable to a study by Rhee et al. [9] using the new titanium plate design without graft which showed a union rate of 77% at 6 months. Our union rate can also be compared to a study by Tanaka et al. [10] which evaluated bone healing in 88 patients who underwent open-door laminoplasty with hydroxyapatite spacers and autogenous graft spacers with union rates on the hinged side of 84% and 79%, respectively, at six months (Table 1).

Although we reported the lowest union rate of 70.5% compared to other studies (Table 1), there was only one study by Rhee et al. that used specific criteria for a reported union rate of 77% which can be compared with our study.

We compared the mean operative time and estimated blood loss of this study with another open-door laminoplasty technique (Itoh and Tsuji) that was performed in the same hospital and with the same surgeon [17]. The mean operative time was significantly less using the maxillofacial plate (181 ± 36 minutes) compared to Itoh and Tsuji's technique (305 ± 46 minutes). However the mean intraoperative blood loss was significantly less in the Itoh and Tsuji group (240 ± 45 mL) compared with the maxillofacial plate (330 ± 82 mL).

Although there was no statistically significant difference in the union rates between levels, the C7 level tended to have the highest union rate at 6 months (83.3%), which may be due to the larger lamina diameter when compared to the other levels.

This study showed that two of 68 levels (2.9%) had evidence of loosening or screw back-out but the maxillofacial titanium miniplate still provided sufficient stability to maintain canal expansion. This may have occurred due to the intact supraspinous and interspinous ligaments and because we applied plates at all levels. There was no study that mentioned the sufficient loads that were needed to maintain canal expansion, but fusion on the hinged side in laminoplasty did not need as much rigid fixation across the motion segments when using spinal fusion procedures.

**Figure 7:**
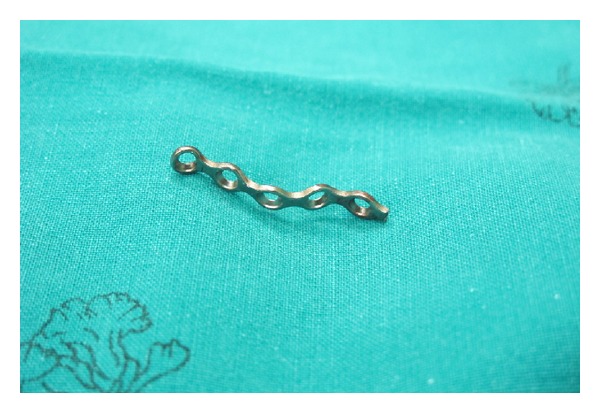
The maxillofacial titanium miniplate can be adjusted in length and bent in all three dimensions to fit the contour of the open-door side of the laminoplasty.

The first advantage of the maxillofacial titanium miniplate over the commercial laminoplasty plate is the cost. The second advantage is that when cases have a bony anatomical variation or the open-door laminoplasty site does not match the commercial laminoplasty plate the maxillofacial titanium miniplate can be adjusted in length and bent in three dimensions to fit the contour of the abnormal anatomy on the open-door side (Figure 7).

A limitation of this study was the 6-month follow-up period. But Wang et al. [15] reported that reclosure of laminoplasty or the “spring-back” phenomenon usually occurs before six months. However a long-term follow-up is needed. Another limitation of this study included not comparing the union rate of the maxillofacial titanium miniplate to other surgical instruments.

## 5. Conclusions

Open-door laminoplasty using a maxillofacial titanium miniplate is a safe and simple fixation technique for the treatment of multiple levels of cervical myelopathy and OPLL. This technique can provide union rates that are comparable to other techniques. A maxillofacial titanium miniplate can maintain canal expansion without failures, dislodgements, or premature closures and is less expensive than the other new titanium miniplate.

## Figures and Tables

**Table 1:** Comparison of the union rates on the hinge side of different types of fixation.

Authors and year	Fixation technique	Graft	Union rate (6 months)
Tanaka et al. [10], 2008	Interconnected porous calcium hydroxyapatite spacer	No	84%
	Autogenous graft spacer	Yes	79%
Rhee et al. [9], 2011*	New titanium miniplate	No	77%
Jiang et al. [11], 2012	New titanium miniplate	Yes	100%
Koopong Siribumrungwong, 2013*	Maxillofacial titanium miniplate	No	70.5%

*Measurement of the union rate with the same specific criteria (Union means bridging of both dorsal and ventral cortices).
